# Screening and quantification of anti-quorum sensing and antibiofilm activity of Actinomycetes isolates against food spoilage biofilm-forming bacteria

**DOI:** 10.1186/s12866-020-02060-7

**Published:** 2021-01-02

**Authors:** Erika Mulya, Diana Elizabeth Waturangi

**Affiliations:** Faculty of Biotechnology, Atma Jaya Catholic University of Indonesia, Jalan Raya Cisauk-Lapan No. 10, Sampora, Cisauk, Tangerang, Banten 15345 Indonesia

**Keywords:** Actinomycetes, Biofilms, *Chromobacterium violaceum*, Food spoilage, Quorum quenching, Quorum sensing

## Abstract

**Background:**

Biofilms can form in many industries, one of them is the food industry. The formation of biofilms in this industry could cause immense economic losses and endanger public health. Biofilms formation is mainly triggered by quorum sensing. Therefore, inhibition of quorum sensing could be an innovative approach to inhibit the formation of biofilms. One way to inhibit quorum sensing is by using anti-quorum sensing compounds. Actinomycetes are a group of bacteria that is acknowledged to produce these compounds.

**Results:**

There were eight crude extracts of Actinomycetes isolates that showed promising anti-quorum sensing activity against *Chromobacterium violaceum*. The concentration of the crude extracts was 20 mg/mL. All the crude extracts showed no antibacterial activity against food spoilage bacteria, except for crude extracts of isolate 18 PM that showed antibacterial activity against *Bacillus subtilis*. They also showed various antibiofilm activity, both inhibition and destruction. The highest inhibition and destruction activity sequentially was done by crude extracts of isolate 12 AC with 89.60% against *Bacillus cereus* and crude extracts of isolate SW03 with 93.06% against *Shewanella putrefaciens*.

**Conclusions:**

Actinomycetes isolates that isolated from different regions in Indonesia can be used as potential candidates to overcome biofilms formed by food spoilage bacteria using their ability to produce anti-quorum sensing compounds.

## Background

Bacteria can communicate with each other using chemical signaling molecules called autoinducers. This communication, known as quorum sensing, is orchestrated in a cell density-dependent manner. Quorum sensing can regulate several activities, such as virulence factor expression, bioluminescence, sporulation, and biofilm formation [1].

Biofilms, a group of bacteria flock together that attach to a surface and enclosed in an extracellular matrix, have significant involvement in various types of industries, one of them is the food industry. The formation of biofilms in this industry can be a firm source of contamination, which responsible for causing food to spoil, which is dangerous for the safety of the food products as well as a massive economic loss. Biofilm also making bacteria more resistant to chemical and physical treatments. Moreover, bacteria that grow as biofilms are hard to remove from the surfaces [2, 3]. There are several bacteria such as *Bacillus cereus*, *Bacillus subtilis*, and *Shewanella putrefaciens* which are known for their ability to form biofilms, thus resulting in food spoilage.

Biofilms usually treated using antibiotics, but this could cause an additional problem as the long-term use of antibiotics might lead to an increase in antibiotics resistance. Furthermore, the use of antibiotics is not suitable in the food industry. Because one of the biofilms formations caused by bacteria quorum sensing, then the inhibition of quorum sensing is expected to inhibit the formation of biofilms. Every activity that inhibits the quorum sensing mechanisms recognized by the name anti-quorum sensing or quorum quenching. Actinomycetes are fungi-like Gram-positive bacteria that are well known to produce a wide range of secondary metabolites from their complex biochemical processes such as anti-quorum sensing and antibiofilm. In a previous study, it reported that *Streptomyces* sp. and *Arthrobacter* sp. crude extracts have antibiofilm activity against *Streptococcus pneumoniae* and *Vibrio parahaemolyticus* [4]. Another study showed that *Streptomyces parvulus* had quorum quenching activity against *Chromobacterium violaceum* and could inhibit biofilm formed by *Pseudomonas aeruginosa*, *Ruegeria* sp., *Staphylococcus aureus*, and *Micrococcus luteus* [5].

It is necessary to conquer the problem of food spoilage caused by biofilms in the food industry. However, research on quorum quenching and antibiofilm activity from Actinomycetes against food spoilage bacteria has not been widely explored.

The objectives of this research are to screen anti-quorum sensing activity of *Actinomycetes* isolates against *Chromobacterium violaceum* as an indicator and quantify their antibiofilm activity against food spoilage biofilm-forming bacteria.

## Results

### Bacterial cultivation

Actinomycetes have some distinct characteristics such as calcareous-like colony, produce earthy odor called geosmin, and strongly attached to the agar medium.

### Screening for anti-quorum sensing activity

A total of 30 Actinomycetes isolates successfully cultivated from cryopreservation. Eight out of 30 isolates (Table 1) used further in this research.

**Table 1:** Actinomycetes isolates and their origin

No	Isolates	Origin
1	1 AC	Pantai Ancol, North Jakarta
2	12 AC	Pantai Ancol, North Jakarta
3	18 PM	Pantai Mutiara, North Jakarta
4	CW02	Cunca Wulang, West Flores (River)
5	SW03	Sawah (Paddy Field) at Gancahan 8 Village, Sleman
6	SW14	Sawah (Paddy Field) at Gancahan 8 Village, Sleman
7	SW16	Sawah (Paddy Field) at Gancahan 8 Village, Sleman
8	KP110	Kulon Progo, Yogyakarta (River)

**Fig. 1:**
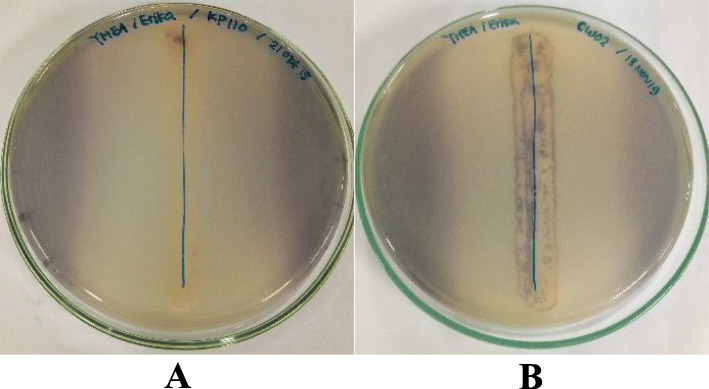
Screening of anti-quorum sensing activity by (**a**) isolate KP110, (**b**) isolate CW02 against *Chromobacterium violaceum*

All eight isolates showed positive anti-quorum sensing activity against indicator bacteria *Chromobacterium violaceum* marked by inhibition of violacein pigment (Fig. 1).

### Antibacterial activity

**Fig. 2:**
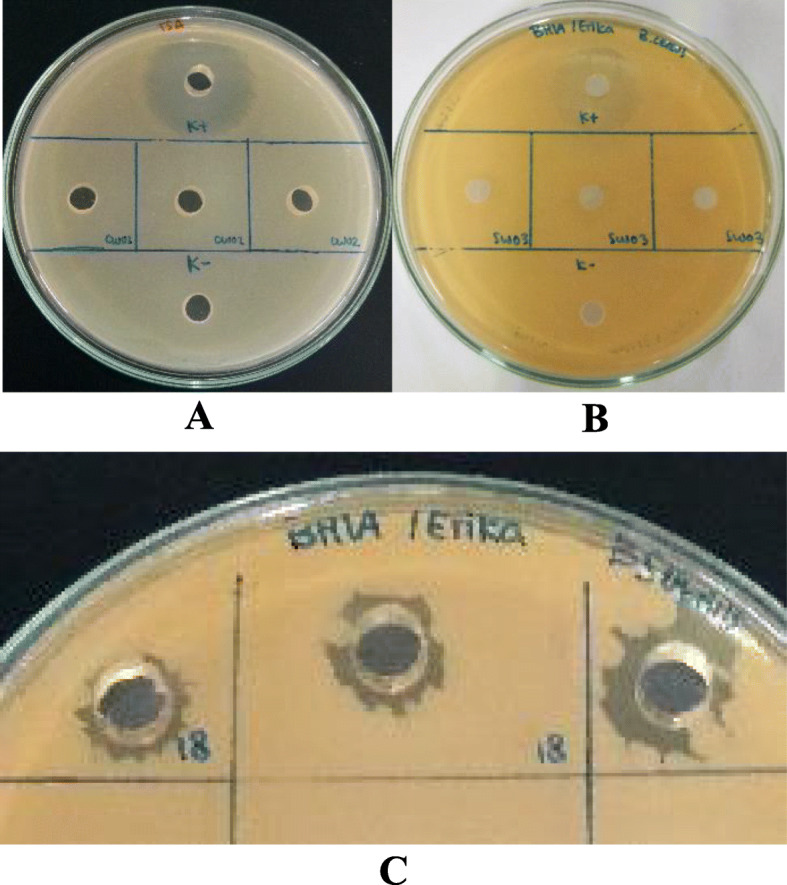
Antibacterial activity of Actinomycetes extracts against (**a**) *Shewanella putrefaciens*, (**b**) *Bacillus cereus*, (**c**) *Bacillus subtilis*

There were 1 out of 8 isolates (18 PM) that showed antibacterial activity against *Bacillus subtilis*. Positive control (Streptomycin) inhibited the growth of food spoilage bacteria (Fig. 2).

### Detection of anti-quorum sensing activity

All eight crude extracts showed positive results. The inhibition performed by each extract was diverse in their activity (Table 2).

**Table 2:** Strength of anti-quorum sensing activity of Actinomycetes crude extracts against *Chromobacterium violaceum*

No	Isolates	Anti-Quorum Sensing Activity
1	1 AC	++
2	12 AC	+++
3	18 PM	+++
4	CW02	+
5	SW03	+
6	SW14	+
7	SW16	+
8	KP110	+

nb: + = weak inhibition, ++ = moderate inhibition, +++ = high inhibition.

**Fig. 3:**
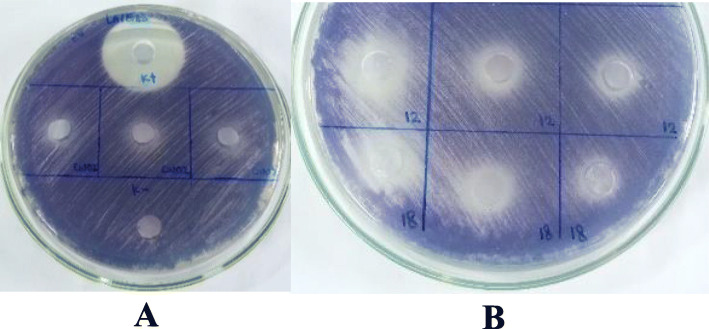
Detection of anti-quorum sensing activity against *Chromobacterium violaceum*

Nevertheless, all of the extracts used have quorum quenching activity against *C. violaceum* (Fig. 3), which was marked by the absence of violacein pigment around the wells.

### Quantification of Antibiofilm activity

All Actinomycetes crude extracts showed both biofilm inhibition and destruction activity against *B. cereus*, *B. subtilis*, and *S. putrefaciens*. The inhibition activity of biofilms formed by the food spoilage bacteria can be seen in Table 3, where biofilms formed by *B. cereus* and *S. putrefaciens* inhibited the highest by crude extracts of isolate 12 AC with inhibitory activity of 89.60 and 87.54%, respectively. Crude extracts of isolate KP110 have the highest inhibitory activity against biofilms formed by *B. subtilis* with 76.43%.

**Table 3:** Biofilm inhibition activity of Actinomycetes crude extracts against food spoilage biofilm-forming bacteria

Pathogens	Inhibition (%)							
	1 AC	12 AC	18 PM	CW02	SW03	SW14	SW16	KP110
*Bacillus cereus*	74.73	89.60	80.52	70.24	55.89	34.12	56.18	78.40
*Bacillus subtilis*	69.76	41.32	X	41.74	54.34	32.98	52.09	76.43
*Shewanella putrefaciens*	84.50	87.54	85.09	24.01	21.70	55.83	82.56	24.18

nb: X = showed antibacterial activity

The destruction activity of biofilms can be observed in Table 4. Biofilms of *B. cereus* eradicated the highest by crude extracts of isolate 1 AC with 75.15%. The highest and lowest destruction activity of *B. subtilis* biofilms are sequentially 93.06 and 79.31% by crude extracts of isolate SW03 and SW16. Crude extracts of isolate 12 AC showed the highest destruction activity against biofilms formed by *S. putrefaciens* with 33.49%.

**Table 4:** Biofilm destruction activity of Actinomycetes crude extracts against food spoilage biofilm-forming bacteria

Pathogens	Destruction (%)							
	1 AC	12 AC	18 PM	CW02	SW03	SW14	SW16	KP110
*Bacillus cereus*	75.15	52.60	72.54	35.02	48.85	71.33	62.13	61.48
*Bacillus subtilis*	86.98	91.90	X	80.70	93.06	86.36	79.31	82.49
*Shewanella putrefaciens*	13.78	33.49	24.96	32.90	22.24	15.40	28.10	31.29

nb: X = showed antibacterial activity

## Discussion

Actinomycetes are widely present both in terrestrial and aquatic environments, where many other bacteria also live in this habitat. Therefore, Actinomycetes need to defend themselves in order to be able to survive and gain nutrients, for example, is by producing various secondary metabolites, such as antibiotics, antifungal, antiviral, anti-quorum sensing, and antibiofilm. Streptomycin, gentamicin, rifamycin, and erythromycin are antibiotics produced by Actinomycetes only [6].

Antibiotics can deal with biofilms formed by bacteria. However, the excessive use of antibiotics can lead to bacterial resistance towards that antibiotics. Besides, the use of antibiotics may be inappropriate in some industries. This has led to the importance of finding other ways to combat biofilm problems. Anti-quorum sensing and antibiofilm compounds from Actinomycetes can be novel to treat biofilms, specifically in this research are biofilms formed by food spoilage bacteria.

*Bacillus cereus* can produce extracellular proteases and lipases that could cause food degradation and food spoilage, thus resulting in a decrease in the food shelf life such as poultry, dairy, and red meat. This bacteria can also induce either emetic or diarrheal gastroenteritis [7]. Similar to *B. cereus*, a sign of *B. subtilis* infection is vomiting accompanied by diarrhea. *B. subtilis* has been reported as a producer of exoproteases and is the major cause of spoilage in bread and other cereal-based food [8, 9], meanwhile *Shewanella putrefaciens* commonly known as spoilage bacteria in seafood, poultry, and beef products that could cause abdominal tract infection and bacteremia [10].

Based on the primary screening of anti-quorum sensing activity, 8 out of 30 isolates are potential as anti-quorum sensing agents as they only inhibit the production of violacein without inhibiting the growth of *Chromobacterium violaceum*. Biochemical assay conducted in a previous study indicated that CW02 was *Arthrobacter* sp., while SW03, SW14, SW16, and KP110 were *Streptomyces* sp.. It also confirmed SW03 to be *Streptomyces* sp. with 96% similarity and the accession number JX434849.

From the antibacterial activity assay, only one crude extract, which is 18 PM that showed positive antibacterial against *Bacillus subtilis*, while the rest showed negative results. Hence, crude extracts of isolate 18 PM will not be used further for antibiofilm activity against related pathogens. This assay was done to prevent a false-positive result. Actinomycetes are recognized as antibacterial producers that instead of inhibiting communication, inhibit the growth of bacteria [11].

Detection of anti-quorum sensing activity was carried out to ensure that all crude extracts used still have anti-quorum sensing activity after extracting. The results showed that all crude extracts still have quorum quenching activity against the indicator bacteria. The signaling molecule of Gram-negative bacteria *C. violaceum* for violacein production is AHL (N-acyl-homoserine lactone). AHL-lactonase, AHL-acylase, paraoxonase, and oxidoreductase are enzymes that can interfere with the mechanisms of AHL quorum sensing [12]. Crude extracts in this research may have those quorum quenching enzymes that disrupt the mechanisms of violacein production. Furthermore, different crude extracts have distinct inhibitory strength, where crude extracts of isolate 12 AC and 18 PM showed the highest inhibition. The amount and type of quorum quenching molecules produced by each isolate may be different, which may explain the difference in inhibitory strength.

The antibiofilm activity was divided into inhibition and destruction activity. Overall, all the crude extracts have both inhibition and destruction activity. Gram-positive bacteria such as *Bacillus* use oligopeptides as their signaling molecules. Npr, PlcR, and PapR are the quorum sensing protein in *B. cereus* that plays a role in biofilms formation [7], while in *B. subtilis* are ComX and CSF (Competence Sporulation Factor) peptides [13]. Gram-negative *S. putrefaciens* use AI-2 (Autoinducer-2) as the quorum sensing signaling molecules [14]. The biofilm inhibition activity may occur because Actinomycetes isolates produce quorum quenching compounds performed in several ways, such as disrupt the signal synthesis, degrade the signaling molecule, obstruct the signal transduction cascade, or hinder the binding of a signal molecule to the receptor [15].

Extracellular Polymeric Substance (EPS) is the major component of the biofilm matrix that responsible for the stability of biofilms that consists of polysaccharides, proteins, lipids, and nucleic acids. The EPS of *B. cereus* contains exopolysaccharides, proteins, and a large amount of extracellular DNA that specifically produce in biofilms [7]. *B. subtilis* matrix includes exopolysaccharides (glucose, galactose, fucose, glucuronic acid, and O-acetyl group) as well as proteins (TasA, TapA, and BslA) [7, 8]. *S. putrefaciens* EPS majorly composed of protein and very low polysaccharide [16]. The biofilm destruction activity may ensue because the Actinomycetes crude extracts contain enzymes that have the ability to degrade EPS, such as glycosidases, proteases, and DNases [17]. A previous study [18] predicted crude extracts of isolate 1 AC and 18 PM as protein or enzyme because they displayed an increase of absorbance value after Proteinase-K and heat loss treatment. The varied activity of inhibition and destruction activity can also cause by assorted factors, for instance, there are still many impurities in the crude extracts, and there might be synergistic or antagonistic activity between the compounds contained in the impure crude extracts, which can increase or decrease the activities [19]. In this research, the exact bioactive compounds and the mechanisms of the crude extracts that performed the anti-quorum sensing and antibiofilm activity, as well as the genus of Actinomycetes isolates used, did not determine.

## Limitation

This study screened for bioactive compounds from Actinomycetes isolates that have quorum quenching activities against *Chromobacterium violaceum* (AHL-based system) based on the reason that AHL is a universal signal that foster inter cell-cell communication. So we do not know whether those isolates have quorum quenching activity against different quorum sensing systems. More research using different indicator bacteria (peptide-based or AI-2-based system) should be performed to determine the exact anti-quorum sensing mechanisms owned by each isolate. We also have not done the characterization of the bioactive compounds, thereby the characterization of each bioactive compound should be carried out in the future to see if the bioactive compounds were mainly composed of proteins. Besides, this study solely provides the preliminary assay of antibiofilm activity, therefore a microscopic-based inhibition of biofilm needs to be done to ensure the biofilm inhibition ability of Actinomycetes crude extracts. Lastly, not all of our isolates have been sequenced, so molecular identification should be executed for all the Actinomycetes isolates.

## Conclusion

A total of 8 Actinomycetes isolates have quorum quenching activity against *C. violaceum* as well as antibiofilm activities both inhibition and destruction against *B. cereus*, *B. subtilis*, and *S. putrefaciens*. Crude extracts of isolates 1 AC, 12 AC, SW03, and KP110 showed promising either for biofilm inhibition and/or destruction activity. This result could contribute to the knowledge of using Actinomycetes as potential candidates to overcome biofilms formed by food spoilage bacteria by their ability as anti-quorum sensing agents. Further research is required to purify the bioactive compounds and characterizes each compound in the crude extracts and to test the crude extracts against different indicator bacteria to determine the mechanisms of the anti-quorum sensing, as well as figure out the mechanisms of biofilms inhibition and destruction for further application.

## Methods

### Bacterial cultivation

Actinomycetes isolates used in this research were obtained from previous studies [20, 21] that isolated from different regions in Indonesia. That previous studies stated that a soil dilution technique using Starch Casein Agar (SCA) was performed for the isolation of Actinomycetes. Pretreatment by heated at 50 °C for 1 h was done for all the sediment samples. Different dilutions (10^− 2^, 10^− 3^, and 10^− 4^) of the marine, river, and paddy field sediment suspensions were shaken under room temperature at 200 rpm for 1 min, then 100 μL of each suspension were spread onto SCA medium and incubated at 28 °C for 7 days. Isolates from that previous studies were grown onto Yeast Malt Extract Agar (YMEA) (Oxoid) and incubated at 28 °C for approximately 7 days. Indicator bacteria *C. violaceum* wild type was cultivated onto Luria-Bertani Agar (LA) (Oxoid) and incubated at 28 °C for 2 days. Food spoilage bacteria *B. cereus* ATCC 10876, *B. subtilis* ATCC 6633 were streaked onto LA and incubated at 37 °C 24 h, meanwhile *S. putrefaciens* ATCC 8071 was streaked onto Tryptone Soya Agar (TSA) (Oxoid) and incubated at 28 °C 24 h. All the bacteria used in this research came from Atma Jaya’s culture collection.

### Screening for anti-quorum sensing activity

The overlay agar method referred to Abudoleh & Mahasneh [22] was used with modifications for this screening. Each Actinomycetes isolate inoculated onto YMEA with a straight streak method and incubated at 28 °C for 2 days. *C. violaceum* was grown separately in 50 mL of Luria-Bertani Broth (LB) (Oxoid) and incubated at 28 °C 24 h. Indicator bacteria then diluted until OD_600_ = 0.132, then a total of 100 μL culture were taken and mixed with semisolid agar (0.75% LA) of 5 mL volume for an overlay on top of the selected Actinomycetes bacteria in the YMEA before, later incubated at 28 °C for 2 days. The positive results were indicated by the absence of violacein pigmentation around the Actinomycetes isolates.

### Extraction of bioactive compounds

This assay referred to Younis et al. [23] with some modifications. Each Actinomycetes isolate cultivated into 100 mL of Tryptone Soya Broth (TSB) (Oxoid) supplemented with 1% glucose and incubated at 28 °C at 120 rpm for 7 days, then transferred to 50 mL sterile conical tubes and centrifuged at 7000 xg for 25 min. The cell-free supernatant was mixed with ethyl acetate (SmartLab) in a 1:1 ratio and then shaken at 120 rpm 24 h. The solvent layer was harvested and evaporated in a rotary evaporator and dried in a vacuum oven at 50 °C. The extracts then weighted, and Dimethyl Sulphoxide (DMSO) 1% was added to achieve the final concentration of 20 mg/mL, later stored at − 20 °C for months.

### Antibacterial activity

Agar well diffusion method based on the research of Tabbouche et al. [24] was used. Food spoilage bacteria (OD_600_ = 0.132) streaked continuously in three different directions, *B. cereus* and *B. subtilis* onto the Brain Heart Infusion Agar (BHIA) (Oxoid) while *S. putrefaciens* onto TSA. After that, wells made using a sterile cork borer. Crude extracts of Actinomycetes (100 μL) then added into the wells and incubated at 37 °C 24 h for *B. cereus* and *B. subtilis* and 28 °C 24 h for *S. putrefaciens*. Streptomycin 10 mg/mL was used as a positive control, while DMSO 1% used as a negative control. Antibacterial activity was observed through a clear zone.

### Detection of anti-quorum sensing activity

Agar well diffusion method referred to Rajivgandhi et al. [25] was used with some modifications. *C. violaceum* (OD_600_ = 0.132) streaked continuously in three different directions onto the LA. Wells were made using a sterile cork borer. The Actinomycetes crude extracts (100 μL) then added into the wells and incubated at 28 °C 24 h. Streptomycin 10 mg/mL was used as a positive control, while DMSO 1% used as a negative control. Quorum quenching activity was observed through the inhibition of violacein production around the wells.

### Quantification of Antibiofilm activity

The quantification of antibiofilm activity was based on the research by Waturangi et al. [4] with some modifications. The activities of antibiofilm were divided into inhibition and destruction activity. For inhibition activity, *B. cereus* and *B. subtilis* inoculated into Brain Heart Infusion Broth (BHIB) and incubated at 37 °C, 120 rpm 24 h, whereas *S. putrefaciens* inoculated into BHIB and incubated at 28 °C, 120 rpm 24 h. A total of 100 μL cultures (OD_600_ = 0.132) and 100 μL crude extract were transferred into a 96-wells microplate and incubated at 37 °C 24 h for *B. cereus* and *B. subtilis* and 28 °C for *S. putrefaciens*. For destruction activity, a total of 100 μL cultures (OD_600_ = 0.132) were transferred into a 96-wells microplate and incubated to form biofilms. After 24 h, 100 μL crude extract was added to each well then reincubated. Overnight culture of food spoilage bacteria without any treatment were used as a positive control, while sterile BHIB was used as a negative control.

After incubation, the planktonic cells and media were discarded and the adherent cells were rinsed twice with deionized water and allowed to air dry. A total of 200 μL of 0.4% crystal violet was used to stain the adherent cells for 30 min. Thereafter, the dye was discarded and the wells were rinsed five times with deionized water and allowed to air dry. Afterward, 200 μL of ethanol was added to solubilize the crystal violet. The optical density was determined at 595 nm with a microplate reader (TECAN M200 PRO). The percentage of inhibition or destruction activity calculated using the following equation:

$$ \%\mathrm{inhibition}\ \mathrm{or}\ \mathrm{destruction}=\frac{\mathrm{OD}\ \mathrm{positive}\ \mathrm{control}-\mathrm{OD}\ \mathrm{sample}}{\mathrm{OD}\ \mathrm{positive}\ \mathrm{control}}\mathrm{x}\ 100\% $$

## Data Availability

The datasets used and/or analyzed during the current study are available from the corresponding author on reasonable request.
